# A novel dietary multi-strain yeast fraction modulates intestinal toll-like-receptor signalling and mucosal responses of rainbow trout *(Oncorhynchus mykiss)*

**DOI:** 10.1371/journal.pone.0245021

**Published:** 2021-01-12

**Authors:** Mark Rawling, Eric Leclercq, Andrew Foey, Mathieu Castex, Daniel Merrifield

**Affiliations:** 1 School of Biological and Marine Sciences, University of Plymouth, Plymouth, United Kingdom; 2 Lallemand SAS, Blagnac, France; 3 School of Biomedical Sciences, University of Plymouth, Plymouth, United Kingdom; University of Illinois, UNITED STATES

## Abstract

This study was conducted to evaluate the mucosal immune responses of rainbow trout when supplementing an experimental formulated feed with multi-strain yeast fraction product (*Saccharomyces cerevisiae* and *Cyberlindnera jardinii*). In total, 360 fish (initial BW 23.1 ± 0.2 g) were randomly allotted into three dietary treatments in an 8-week feeding trial. The dietary treatments included basal diet (control) and control + 1.5 g/kg multi-strain yeast fraction product (MsYF) fed continuously and pulsed every two weeks between control and MsYF diet. No negative effects on growth performance of feeding the MsYF supplemented diet were observed. SGR and FCR averaged 2.30 ± 0.03%/day and 1.03 ± 0.03, respectively, across experimental groups. Muscularis thickness in the anterior intestine after 8 weeks of feeding was significantly elevated by 44.3% in fish fed the MsYF continuously, and by 14.4% in fish fed the MsYF pulsed (*P* < 0.02). Significant elevations in goblet cell density in the anterior and posterior (>50% increase) intestine were observed after 8 weeks of feeding the MsYF supplemented diet (*P*< 0.03). In contrast, lamina propria width was significantly lower in fish fed the experimental diets (>10% reduction). The gene expression analysis of the intestine revealed significant elevations in expression of *tlr2*, *il1r1*, *irak4*, and *tollip2* after 4 weeks of feeding the MsYF. Significant elevations in effector cytokines *tnfα*, *il10* and *tgfβ* were observed after 4 weeks of feeding the MsYF regime. After 8 weeks significant elevations in the gene expression levels of *il1β*, *ifnγ*, and *il12* were observed in fish fed the MsYF. Likewise, the expression of the transcription factor *gata3* was significantly elevated (*P*<0.01). Supplementation of the multi-strain yeast fraction product positively modulates the intestinal mucosal response of rainbow trout through interaction with toll-like receptor two signalling pathway and potential for increased capacity of delivery of antigens to the underlying mucosal associated lymphoid tissue.

## Introduction

With increased awareness of antibiotic resistant bacterial strains, the aquaculture industry is increasingly integrating functional health feeds as a preventative health and welfare management strategy. Indeed to minimize the use of veterinary drug usage significant interest in finding alternative in feed solutions such as using feed supplements from yeast cell wall extracts have been widely studied as a strategy to improve animal welfare and health [1]. Among the range of yeast-based products, extracts from the cell wall of *Saccharomyces cerevisiae* have received much research focus across aquaculture species validating their subsequent inclusion and contribution to improving animal health and welfare [2,3]. Many studies have concluded positive benefits of inclusion of yeast cell wall extracts such as β-glucans and mannan-oligosaccharides at doses of inclusion ≤20 g/kg to fish mucosal associated lymphoid tissues (MALT) [3–6]. Recently, in teleosts as a result of controlled trials using an *in vitro* approach to studying the effects of innate immune cells exposed to β-glucans it has become apparent that innate cell responses can be differentiated to tolerant or trained phenotypes, providing a resource to further understand and manipulate immune-mediated responses [7]. The notion of ‘trained immunity’ will become an important focus in future investigations into how MALT is affected by the different yeast cell wall extracts.

The mucosal associated lymphoid tissues of teleost fish are the first line of defence that involves the recognition and processing of invading pathogens, sensing of self and non-self antigens and regulation of immune responses through germline encoded pathogen recognition receptors (PRRs). These PRRs can bind conserved and invariant structures called pathogen associated molecular patterns (PAMPs). Yeast cell walls (YCWs) contain a number of other recognised ligands called microbial associated molecular patterns (MAMPs) including β-glucans and α-mannans that show well-documented positive effects in teleost fish [2,7,8]. Indeed, the activation through different PRRs present on innate immune cells effectively allow the host to determine the immune fate of localised gut associated lymphoid tissues (GALT) by allowing for tolerance of safe non-self antigens, such as commensal microbes and food antigens. Nevertheless, maintaining the ability to augment an inflammatory response toward unsafe non-self, pathogenic material [9,10]. In mammals, the innate recognition of yeast MAMPs is achieved by PRRs present on macrophages and dendritic cells (DC) amongst other innate immune cells, which are specialised phagocytic and antigen presenting cells that govern innate and adaptive immune responses mounted by mucosal tissues [11,12]. In teleosts, macrophages and dendritic cells play a similar important role in recognition and augmentation of the immune responses that are fundamental to the homeostatic functions of the gastrointestinal tract [13]. In mammals and teleosts, PPRs from the Toll-like receptor (TLR) and C-type lectins (CLR) families are important in the recognition of yeasts and YCW fractions such as mannans and β-glucans [14–16]. Specifically, 22 recognised TLRs play an important role in recognising yeast ligands such as zymosan, phospholipomannan, *O*-linked mannans, glucoronoxylomannan, and fungal DNA [17–19].

The use of YCW extracts from *Saccharomyces cerevisiae* to positively modulate the mucosal immune responses through tolerance to commensal organisms or immune responsiveness to extrinsic factors have been well documented in aquatic and terrestrial livestock [7,20,21]. However, yeast cell wall (YCW) fractions from non-Saccharomyces are not as well documented. YANG^®^ is a new generation multi-strain yeast fraction (MsYF) combining the use *S*. *cerevisiae* and a non-saccharomyces strain *Cyberlindnera jadinii*. *C*. *jadinii* was recently identified as a probiotic antagonist to the human fungal pathogen *Candida albicans* [22]. Furthermore, *C*. *jadinii* is a close relative of *Candida utilis* (also referred to as *Torula* yeast), which has been used in the biotechnology industry for many applications [23]. Initial investigations using human monocytes *in vitro* identified that exposure to the MsYF increased the production of the pro-inflammatory cytokine TNF-α and respiratory burst capacity. Furthermore, analysis of the glycoprotein structure showed a higher degree of longer unfolded glycoproteins presented by MsYF compared to a single strain yeast fraction, which could confer more sites for interaction with host innate immune cells [24]. Previously, our group reported that dietary supplementation with the MsYF at a dose below 1g /kg feed modulated the intestinal expression of specific genes markers of intestinal health and immunity, increased the intestinal surface area, i.e. microvilli density, and enhanced the growth (SGR) and feed (FCR) performance in the European seabass (*Dicentrarchus labrax*). To date little is known about the effects of feeding the MsYF on the intestinal mucosal responses of freshwater fish.

## Materials and methods

### System and fish

Experimentation was carried out at the Aquaculture and Fish Nutrition Research Aquarium, University of Plymouth (Plymouth, UK) within an indoor freshwater recirculated aquaculture system (RAS) equipped with mechanical and biological filtration, UV-disinfection, photo-and-thermo control, and aeration. The RAS system consisted of 9 rectangular fibreglass tanks (135/ l, central drain) provided with a water flow rate at 900 l/hr/tank in a circular flow motion. Rainbow trout fingerlings were sourced locally from Exmoor fisheries (Exmoor, UK) received a general purpose prophylactic treatment on arrival, were quarantined and acclimatised for four weeks then randomly distributed into the experimental system (9 tanks with 30 fish/tank, initial mean body-weight (BW) = 23.1 ± 0.2 g) at the beginning of the trial. During the trial, fish were kept under a constant 12:12 hr light:dark photoperiod and water quality parameters were maintained within a suitable range for rainbow trout [25] as follows: water temperature = 14.5 ± 0.5°C, pH = 6.8–7.5, dissolved oxygen = 7.5–8 mg/ l, ammonium = 0.04–0.08 mg/ l, nitrite = 0.02–0.06 mg/ l and nitrate = 54–58 mg/ l. Animals were investigated and handled in accordance with the Animals (Scientific Procedures) Act 1986 (ASPA) revised to transpose European Directive 2010/63/EU as currently in force since 1 March 2014 in England. The trial and procedure applied were reviewed and approved by the University of Plymouth animal welfare and ethical review board (AWERB).

### Experimental diets

A basal diet was formulated using feed formulation software (Feedsoft^®^) to contain 45% crude protein and 20% crude lipid as per the known nutritional requirements of juvenile rainbow trout [26]. The test diet was then produced by supplementing, prior to cold extrusion, the required amount of basal diet with the multi-strain yeast fraction product (MsYF, Lallemand SAS, Blagnac, France) at 1.5 g/kg of feed (Table 1). The composition and structure of each yeast strain fraction present in the MsYF product was reported previously [24]. The diets were produced by mechanically stirring the ingredients into a homogenous mixture using a Hobart food mixer (Hobart Food Equipment, Australia, model no: HL1400— 10STDA mixer). Warm water was added to reach a consistency suitable for cold press extrusion to form 1 mm pellets (PTM Extruder system, model P6, Italy). The nutritional profile of the diets (Table 1) were determined according to AOAC protocols [27].

**Table 1:** Formulation (g/kg) and proximate composition of experimental diets.

	Control	MsYF
**Feed commodity**		
Fishmeal LT94^1^	300.0	300.0
Soybean meal dehulled^2^	100.0	100.0
SPC60^3^	143.0	143.0
CGM^3^	40.0	40.0
Vital wheat gluten	100.0	100.0
Fish oil^4^	82.5	82.5
Rapeseed oil	80.0	80.0
Corn starch^5^	139.5	139.5
Vitamin +mineral^6^	10.0	10.0
CMC binder^4^	5.0	5.0
MsYF		1.5
**Proximate analysis**		
Dry matter (DM; %)	95.8	95.6
Crude Protein (% DM)	47.2	47.4
Crude Lipid (% DM)	19.0	19.9
Ash (% DM)	7.0	7.4
Energy MJ/ kg	22.1	22.3

^1^United fish products (Aberdeen, Scotland, UK).

^2^HP-110, Hamlet Protein, UK (crude protein 57.5%; ash 6.8%; moisture 6.5%; lipid 2.5%).

^3^Skretting feed ingredients (Stavanger, Norway).

^4^Epanoil (Seven Seas Ltd, UK).

^5^Sigma-Aldrich (Poole, UK).

^6^Premier nutrition vitamin premix (Calcium 12.1%, magnesium 1.6%, phosphorous 0.5%, vit A 1.0μg/kg, vit D3 0.1 μg/kg, vit E (as alpha tocopherol acetate) 7,000 mg/kg, copper (as cupric sulphate) 250.0 mg/kg, ash 78.7%.

### Experimental design and feeding

Rainbow trout were fed one of three dietary regimes in triplicate tanks (9 tanks): 1) Control (Basal diet), 2) MsYF diet continuously fed (MsYF_C) or 3) MsYF diet pulsed fed (MsYF_P) with 2 weeks on MsYF diet and 2 weeks on control diet until trial’s completion. Fish were hand-fed a regime of 3% ± 0.6% biomass per day distributed in three equal daily meals (0900, 1300 and 1700 hrs). Biomass per tank was estimated daily based on predicted growth rate and adjusted bi-weekly by bulk-weighting following a 24 h starvation period.

### Sampling schedule

After 4 and 8 weeks of feeding (trial mid- and end-point), a total of 6 and 12 fish per experimental group, respectively, were randomly netted and euthanized following Home Office schedule 1 procedures (UK). Fish were individually measured for body-weight (BW; ± 0.1 g); fork-length (FL; ± 1 mm) and dissected. For histological analysis, anterior (AI) and posterior intestinal (PI) samples were excised, washed from digesta using phosphate buffer saline (pH 7.2, Sigma Aldrich, UK), fixed in formalin at 4°C for 48 h then stored in 70% ethanol until processing. For scanning electron microscopy (SEM), PI samples were washed in 1% S-carboxymethyl-L-cysteine buffer (pH 7.2) and preserved in 2.5% glutaraldehyde with 0.1 M sodium cacodylate buffer (1:1 v/v, pH 7.2) until processing. For gene expression analysis (n = 2 per tank, n = 6 per treatment), PI samples (<100 mg) were placed into 1 mL RNA-later solution (Applied Biosystems, UK); stored at 4°C for 24 h then at -80°C until RNA extraction.

### Growth and feed performance calculations

For growth performance assessment, 30 fish/tank were bulk-weighed on a bi-weekly basis. Growth and feed performance were assessed based on specific growth rate (SGR), feed conversion ratio (FCR) and Fulton’s condition factor (K) calculated as follow: SGR (%BW/day) = 100((lnBWf—lnBWi)/T); FCR = FI /WG and K = BW/ FL^3^; where BWf = final body-weight (g), BWi = initial body-weight (g), T = duration of the trial (day), WG = weight-gain (g), FI = feed input (g) and FL = fork-length (cm).

### Intestinal morphometry by light-microscopy and scanning electron microscopy (SEM)

Formalin-fixed AI and PI samples were dehydrated, embedded in paraffin wax, sectioned at 5 μm thickness and dried in an oven overnight at 37°C. For each specimen, multiple sections were stained with Haematoxylin combined with Alcian Blue and van Gieson (AB-vG) to assess the muscularis thickness, mucosal fold length, laminar propria width and goblet cell density in the epithelium after Dimitroglou and colleagues [28]. Image analysis was conducted using Image ‘J’ 1.47v software (National Institutes of Health, USA), and a high through-put algorithm was developed to count goblet cells in image ‘J’, based on threshold capture of specific goblet cells. SEM samples were processed according to methodologies outlined previously by Rawling and colleagues [24].

### Intestinal RNA extraction and cDNA synthesis

Total RNA was extracted using TRI reagent (Ambion, Life technologies, UK) according to the manufacturer’s instructions, with some modifications. Briefly, 50–100 mg posterior intestinal samples were removed from the RNAlater solution and excess solution was removed by pressing the sample between sterile tissue. Samples were then transferred into a tube containing 1 mL TRI reagent and ceramic beads and homogenised for 40 secs using FastPrep-24 5^G^ machine following the manufacturer’s instructions (MP Biomedicals, Europe). The resulting supernatant was transferred into a 2 ml Eppendorf tube, 200 μl of chloroform was added, samples were then vortexed then centrifuged at 12,000 x g for 15 min. The upper aqueous phase was transferred into a tube containing an equal volume of isopropanol. Mixtures were vortexed and centrifuged at 14,000 x g for 15 min. Supernatants were discarded and the precipitated RNA pellets were washed using 1 ml of 75% ethanol. Total RNA was dissolved in diethylpyrocarbonate (DEPC) and to remove any contaminating genomic DNA were purified using RNeasy Plus Mini Kit according to the manufacturer’s instructions (Qiagen, UK). The concentration and quality of RNA in each sample were determined by measuring 260/280 nm and 260/230 absorbance ratios (NanoDrop Technologies, Wilmigton, USA). The integrity of RNA was confirmed by running samples on a 1% agarose gel, samples were stored at -80°C. A total amount of 1 μg of RNA was used for cDNA synthesis, employing iScript cDNA synthesis kit (Bio-Rad, UK). The reaction was placed at 25°C for 5 min, then 42°C for 30 min and inactivated at 85°C for 5 min. The iScript cDNA synthesis kit contains a combination of oligo dTs and random hexamers to work with a wide variety of targets.

### Real-time PCR assay

PCR reactions were performed with SYBR green method using a StepOne Plus™ Real time-PCR and the Quant studio thermal cycler (Applied Biosystems). Duplicate PCR reactions were carried for each sample analysed. Each PCR reaction was set on a 384 well plate by mixing 2 μl of diluted (1/10) cDNA with 5.5 μl 2 x concentrated iQ™ SYBR Green Supermix (Bio-Rad), containing SYBR Green as a fluorescent intercalating agent, 0.3 μM forward primer and 0.3 μM reverse primer. The primer used and their sequences are presented in Table 2. The thermal profile for all reactions was 10 min at 95°C and then 40 cycles of 15s at 95°C, 60s at 59°C. Florescence monitoring occurred at the end of each cycle. Additional dissociation curve analysis was performed and showed in all cases one single peak. *β-actin* and *elf1-α* were used as reference genes in each sample in order to standardise the results by eliminating variation in mRNA and cDNA quantity and quality [29]. The stability and suitability of *β-actin* and *elf1-α* as reference genes were confirmed according to the algorithms used by geNorm™ software [30]. An expression stability value ‘M’ was generated for reference genes. No amplification product was observed in negative controls and no primer-dimer formations were observed in the control templates. Modification of gene expression is represented with respect to the controls being sampled at the same time as the treatment.

**Table 2:** Primer pair sequences, gene name abbreviations, annealing temperature (Aneal Tm in°C), amplicon size (bp) and primer efficiency (Eff) for genes used for real-time PCR.

Gene name	Primer name	Accession number	Primer Sequence (5’-3’)	Aneal Tm/Amplicon/Eff
Elongation factor 1-alpha	*elf1-α* Fwd	KC747822.1	TGCGGAGGCATTGACAAGAG	60/92/2.1
	*elf1-α* Rev		TCCAGCACCCAGGCATACTT	
β-Actin	*β-actin* Fwd	AJ438158.1	AGCCCTCCTTCCTCGGTATG	60/81/2.1
	*β-actin* Rev		GGATGTCCACGTCACACTTCAT	
Toll-like receptor 2	*tlr2* Fwd	NM_001124419.1	TCTTTGGAGAGGATGGGTATGG	60/92/2.1
	*tlr2* Rev		GCCTTGACCCTCTCTTCACTA	
Myeloid differentiation gene 88	*myd88* Fwd	NM_001124421.1	CCATCACCAGCGAACTCATC	60/80/2.1
	*myd88* Rev		GGCATCACTGTCCAGGTACT	
Interleukin 1 receptor, type I	*il-1r1* Fwd	AJ295296.1	CGGAGAAGCAGACGACTCAT	60/93/2.1
	*il-1r1* Rev		GCTCTGGTGCAGTGGTAACT	
Interleukin-1 receptor-associated kinase 4	*irak4* Fwd	FN598575.1	CCGAGGTACTCTCAGCAACAT	60/112/2.2
	*irak4* Rev		CTCCCACGGTGCAGTTAGAT	
Toll-interleukine I receptor interacting protein II	*tollip2* Fwd	AJ878917.1	GGAATCCCTGGGCACTGTAA	60/89/2.1
	*tollip2* Rev		AAGGGTCCATGCGTGTCATA	
Interleukin-1-beta	*il-1β* Fwd	NM_001124347.2	GGACATGCAGCAGGACTACA	60/83/2.0
	*il-1β* Rev		GCTGGATGGTGAAGGTGGTA	
Tumour necrosis factor alpha	*tnf-α* Fwd	NM_001124357.1	AGCCCTACTCTTTGCATGGT	60/81/1.9
	*tnf-α* Rev		GCACCAATGAGTATCTCCAGTT	
Interleukin-10	*il-10* Fwd	NM_001245099.1	GCTGGACGAAGGGATTCTACA	60/89/2.1
	*il-10* Rev		GCACCGTGTCGAGATAGAACT	
Transforming growth factor beta	*tgfβ* Fwd	X99303.1	CCCACTGGCTACTTTGCTAAC	60/95/2.1
	*tgfβ* Rev		TGCTTATACAGAGCCAGTACCT	
Interleukin-12	*il-12* Fwd	HE798148.1	CAGTGAGAGTGCGTGTCTGA	60/80/2.0
	*il-12* Rev		CGGCCTGTTTGTAAGCCTGTA	
Interferon Gamma	*ifn-γ* Fwd	NM_001124620.1	GACAGTGAGCAGAGGGTGTT	60/80/2.1
	*ifn-γ* Rev		CCCGTCTGGTTCAGCATCTG	
T-box transcription factor 21	*t-bet* Fwd	FM863825.1	CGCAGACATCACCCAGCTAA	60/90/2.1
	*t-bet* Rev		GAGTCAGGTGGTGCGTACAG	
Signal transducer and activator of transcription 6	*stat6* Fwd	HG794521.1	CGTTCCCTGGAAGCAGATGT	60/103/2.1
	*stat6* Rev		TTGGGCCAGGAAATGTTGGT	
GATA-binding protein 3	*gata3* Fwd	NM_001195792.1	ACCTCGGCCACTCGTACAT	60/87/2.1
	*gata3* Rev		GGTTGCCCTGTGAGTCGATA	

The threshold cycle (Ct), defined as the point at which the fluorescence rises appreciably above the background fluorescence, was determined manually for each run. PCR efficiencies for each set of primers were determined using 10-fold serial dilutions of cDNA (n = 3) and resulting plots of Ct versus the logarithmic cDNA input were used to calculate the efficiencies using the equation E (PCR efficiency) = 10(-1/slope) after Rasmussen [30] (Table 2). The expression of target genes (FC (Log^2^) were calculated on the basis of Ct deviation (ΔCt) of the unknown sample versus a control sample, and expressed in comparison to the reference genes *β-actin* and *elf1-α* according to calculations outlined by geNorm™ manual (http://medgen.ugent.be/~jvdesomp/genorm/) and Vandesompele and colleagues [31].

### Statistical analysis

All statistical analyses were carried out using R version 3.4.1 [32]. Rt-qPCR data were analysed using the permutation after Ohmel [33]. All other data were assessed by one-way ANOVA tests with Tukey HSD post-hoc test was used to show where differences in experimental groups. Significance was accepted at *P* < 0.05. Data are presented as mean ± standard deviation (SD).

## Results

### Growth performance

There was no significant difference in body-size parameters between treatments at the beginning, mid and end-point of the trial. Over the trial duration, SGR and FCR averaged 2.30 ± 0.03%/day and 1.03 ± 0.03, respectively, across experimental groups (Table 3).

**Table 3:** Growth performance of rainbow trout over the 8-week trial’s duration (n = 3 tanks/ treatment).

	Control	MsYF_C	MsYF_P	*P-*value
Initial body-weight (g)	23.1 ± 0.3	23.1 ± 0.3	23.1 ± 0.2	0.973
Final body-weight gain (g)	83.3 ± 2.9	80.0 ± 2.1	82.2 ± 1.5	0.218
Fulton’s condition factor	1.46 ± 0.1	1.43 ± 0.1	1.41 ± 0.1	0.092
Feed conversion ratio (FCR)	1.00 ± 0.01	1.05 ± 0.1	1.03 ± 0.02	0.175
Specific growth rate (SGR,%/day)	2.34 ± 0.04	2.28 ± 0.1	2.29 ± 0.06	0.156

Data presented as mean ± SD (n = 3 tanks / treatment).

### Gut morphometry

**Fig 1:**
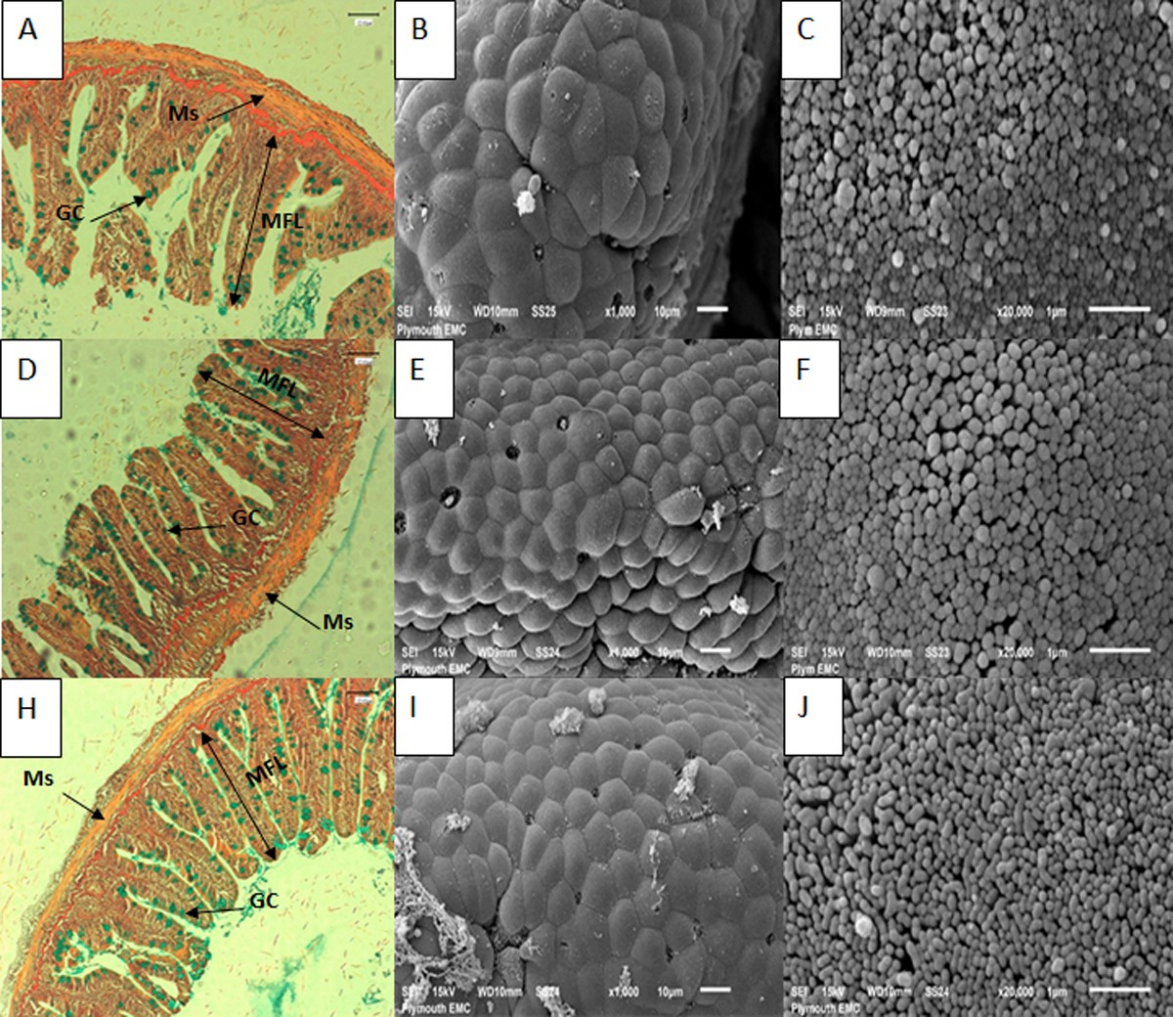
Photomicrographs and scanning electron micrographs of posterior intestine of rainbow trout indicating no signs of damage to tissues when fed with experimental and control diets. Images A-C represent control fed fish, A) cross section of intestine with AB-vG staining (x40 objective, scale bar is 100 μm), arrows indicate different structures including: goblet cell (GC), muscularis (Ms) and mucosal fold length (MFL); B) scanning electron micrograph showing section of posterior intestine (scale bar is 10 μm); C) scanning electron micrograph of microvilli of intestine (scale bar is 1 μm). Images D-F represent fish fed MsYF continuously; Images H-J represent fish fed MsYF pulsed with control every 2 weeks.

The authors measured two regions of the intestine to access the effects of the MsYF on both the anterior intestine (AI), where the function of the enterocytes can be considered as absorptive cells, and posterior intestine (PI) where enterocytes are characterised by antigen sensing and uptake [34,35]. Rainbow trout intestine showed no signs of necrosis or enteritis like pathologies (Fig 1). With regard to mucus integrity of the intestinal lumen, goblet cell density compared to the control was significantly elevated in the MsYF continuously fed group by 4.7% in the anterior intestine and 53.9% in the posterior intestine. Likewise, in the MsYF pulsed fed group there was a significant elevation by 15.4% in the anterior and 58.0% in the posterior intestine (Table 4). Further, in the anterior intestine a significant elevation in muscularis thickness and a significant reduction in laminar propria width were observed compared to the control in both continuously (+44.3% and -11.4%, respectively) and pulsed (+14.4% and -21.7%, respectively) fed MsYF groups. There were no other differences in intestinal morphometry between experimental groups (Table 4).

**Table 4:** Morphometric data from anterior and posterior intestine of rainbow trout fed experimental diets for 8 weeks.

	Control	MsYF_C	MsYF_P	*P*-value
**Anterior intestine**				
Muscularis thickness (μm)	37.3 ± 7.1^a^	53.9 ± 15.8^b^	42.7 ± 10.2^c^	<0.02
Laminar propria width (μm)	23.2 ± 3.1^a^	20.6 ± 2.6^b^	18.2 ± 2.8^c^	<0.002
Mucosal fold length (μm)	434.9 ± 97.9	457.6 ± 91.7	465.1 ± 62.6	0.934
Goblet cell density (n/1000 μm)	150.7 ± 40.0^a^	158.1 ± 32.2^b^	178.1 ± 33.9^b^	<0.02
**Posterior intestine**				
Muscularis thickness (μm)	37.3 ± 10.8	40.7 ± 6.5	41.6 ± 10.8	0.934
Laminar propria width (μm)	19.3 ± 3.2	19.3 ± 2.7	17.9 ± 3.2	0.999
Mucosal fold length (μm)	530.8 ± 127.6	511.6 ± 91.7	502.7 ± 102.5	0.967
Goblet cell density (n/1000 μm)	33.9 ± 13.4^a^	73.6 ± 18.7^b^	80.7 ± 33.5^b^	<0.001

Data is presented as means ± SD (n = 12/ treatment). Super scripts ^a-c^ show significant differences between fish fed control and experimental diets.

### Intestinal gene expression

**Fig 2:**
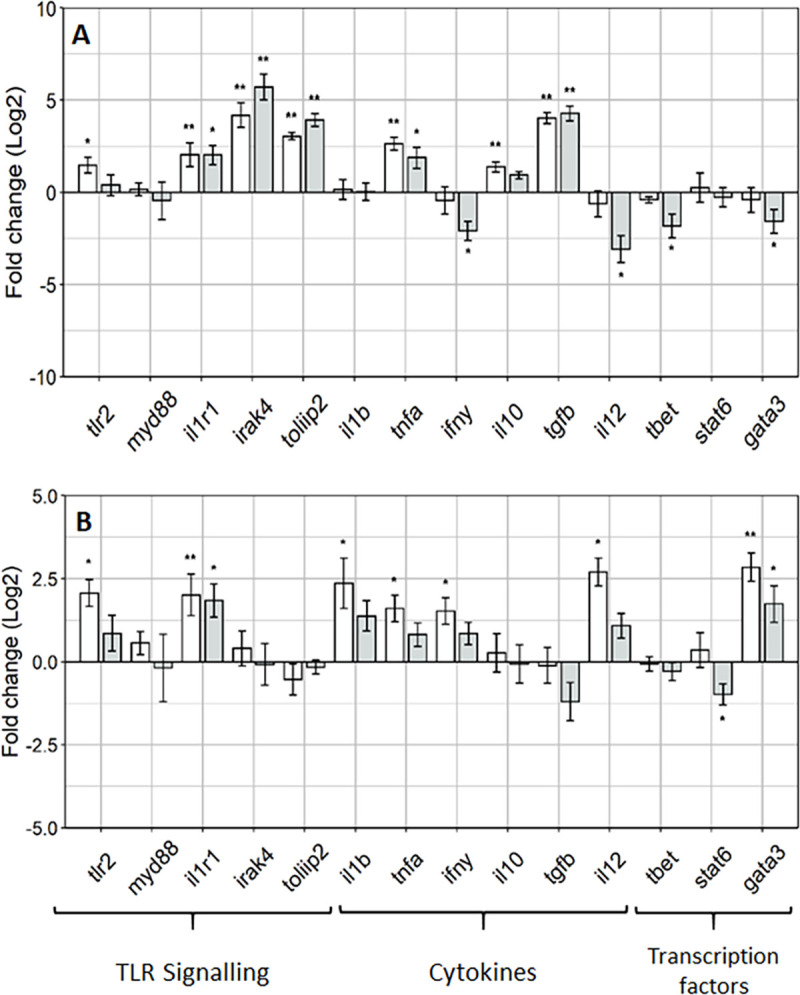
Rainbow trout posterior intestine show regulation of innate and adaptive responses through induction of TLR signalling. Gene expression profiles posterior intestine of rainbow trout at week 4 (A) and 8 (B) relative to the Control (dotted line). Data presented mean ± SEM (n = 6 fish per treatment; Fold change(log2)); light grey bars indicate fish fed MsYF continuously, dark grey bars indicate fish fed MsYF pulsed with control every 2 weeks. Asterisks denote significant differences between treatment and control groups: * = *P* < 0.05; ** = *P* < 0.01.

The focus of the gene expression analysis was on the posterior intestine as enterocytes in this region show important features for antigen sensing and uptake such as an irregular microvilli zone and high pinocytotic activity at the apical part [34]. The aim of the investigation was to access the effects of a novel MsYF on the intestinal mucosal responses of rainbow trout, and here the authors focus on gene expression targets for the intestinal cellular TLR signalling pathways. Overall, the investigation revealed upregulation in the gene expression targets for toll-like receptor 2 signalling, effector cytokines and transcription factors in fish fed the MsYF compared to control. After 4 weeks, fish fed the MsYF continuously regime revealed a significant >2 fold increase in expression of all TLR signalling targets (Fig 2A), where elevations were observed in *tlr2* by 57% (*P* = 0.019), *il1r1* by 70% (*P =* 0.002), *irak4* by 92% (*P* = 0.004) and *tollip2* by 88% (*P* = 0.002). Similarly, fish fed the pulsed regime revealed significant >2 fold elevations in the expression of *il1r1* by 71% (*P* = 0.02), *irak4* by 97% (*P* = 0.002) and *tollip2* by 94% (*P* = 0.002), compared to control. Pro-inflammatory cytokine *tnfα* was significantly elevated in both continuously and pulsed fed MsYF groups by 83% (*P* = 0.002) and 70% (*P* = 0.01), respectively. No differences were observed in pro-inflammatory cytokine *il1β* expression across all treatment groups. In the continuously fed MsYF group anti-inflammatory cytokines *il10 and tgfβ* revealed significantly elevated expression by 60% (*P* = 0.006) and 95% (*P* = 0.002), respectively. In the pulsed fed MsYF group revealed a significant elevation by 95% (*P* = 0.002) in the expression of *tgfβ* (Fig 2A). In contrast, fish fed the MsYF pulsed revealed significant >2 fold down regulation in the expression in *il12* by 875% (*P* = 0.05) and *ifnγ* by 401% (*P* = 0.03), compared to the control group. Likewise, the MsYF pulsed group revealed significant down regulations by 146% (*P* = 0.02) and 246% (*P* = 0.05) in the expression of transcription factors *t-bet* and *gata3*, respectively.

After 8 weeks, there were significant elevations in the expression of TLR signalling targets *tlr2* by 70% (*P* = 0.05) and *il1r1* by 68% (*P* = 0.006) in fish fed the MsYF continuously. Whereas in the MsYF pulsed fed group revealed a significant upregulation in the expression of *il1r1* by 70% (*P* = 0.03), compared to control group. The expression of pro-inflammatory cytokines revealed significant elevations in *il1β* by 72% (*P* = 0.02) and *tnfα* by 64% (*P* = 0.05) in the MsYF continuously fed group. Moreover, significant elevations were observed in the expression of *ifnγ* by 69% (*P* = 0.04) and *il12* by 86% (*P* = 0.02) in the MsYF continuously fed regime, compared to control group. The expression levels of transcription factor *gata3* revealed a significant elevations in fish fed the MsYF continuously by 86% (*P* = 0.008) and by 69% (*P* = 0.03) in MsYF pulsed, compared to the control regime (Fig 2B. In contrast, a significant down regulation was observed in the expression of *stat6* by 81% (*P* = 0.03) in fish fed the MsYF pulsed.

## Discussion

The aim of the study was to investigate the effect of dietary supplementation with a MsYF product combining two strains of *S*. *cerevisiae* and a single strain of *C*. *jardinii* on the intestinal mucosal immune responses of rainbow trout fingerlings under non-challenging conditions. Results indicate that dietary supplementation with the MsYF significantly altered the intestinal expression profiles of genes associated with the innate and adaptive immune responses and histo-morphometry.

Fungal cell walls contain numerous glycans, glycolipids and glycoproteins collectively known as MAMPs, as reviewed elsewhere by Erwig and Gow [36]. Indeed the presentation of fungal MAMPs are recognised by a plethora of PRRs present on phagocytes of the innate immune system including Toll-like receptors (TLRs), C-type lectin receptors (CLRs) and to a lesser extent NOD- like receptors (NLRs) [37–39]. In teleosts, many studies have identified that TLRs and NLRs are present [40,41], however there is much debate as to whether C-type lectin receptors exist in fish [42]. Accordingly, in the present study the authors present data for the TLR-mediated signalling pathway to identify the recognition of MAMPs presented by the inclusion of the MsYF. In the posterior intestine significant elevations in *tlr2*, interleukin 1 receptor, type I (*il1r1*), interleukin-1 receptor-associated kinase 4 *(irak4)* and toll interacting protein (*tollip2*) expression were observed after 4 weeks of feeding the MsYF supplementation, relative to the control. In particular, the results indicate a highly significant elevation in *irak4* (>2 fold increase), which is central to the TIR domain and subsequent TLR signalling cascade. Other studies using rainbow trout *irak4* report that although the structure of *irak4* is very similar to the mammalian *irak4* the function of teleost *irak4* can impair the TLR signalling in human HEK-293 cells *in vitro* [43]. Negative regulation of TLR mediated signalling is important to confer balance of immune-responsiveness and tolerance. Indeed, Smythies and colleagues reported that during maturation from monocytes, human intestinal macrophages down regulate key TLR signalling molecules such as myeloid differentiation primary response 88 (MyD88) and tumor necrosis factor receptor (TNFR)-associated factor 6 (TRAF6), and up-regulate negative regulators such as interleukin-1 receptor-associated kinase m (IRAK-M) and A20 [44,45]. The current study shows evidence that low expression of *myd88* and robust gene expression of both a 3-fold increase in *tollip2* and 4.2-fold increase in *irak4* after 4 weeks of feeding the MsYF continuously. Moreover, the significant 3.9-fold increase in expression of *tollip2* and 5.7-fold increase in the expression of *irak4* in the MsYF pulsed fed fish, maybe an indication of negative regulation of TLR signalling in fish fed the MsYF similar to how TOLLIP and IRAK-M in mammalian cells impair TLR signalling [46]. Negative regulation of TLR signalling is important because prolonged and excessive activation of TLRs can lead to uncontrolled inflammation detrimental to the host [47–49].

In mammals, many *in vitro* studies have reported that macrophages and DCs exposed to yeast MAMPs display cytokine profiles characteristic of a more balanced pro-inflammatory versus anti-inflammatory response [50–52]. In the current study in comparison to the control group, MsYF supplementation revealed significant elevations in the expression profiles of both pro-inflammatory tumor necrosis factor alpha and interleukin 1 beta (*tnfα*, *il1β*) and anti-inflammatory interleukin 10 and transforming growth factor beta (*il10*, *tgfβ*) cytokines demonstrating a balance between immune responsiveness and mucosal tolerance. This result agrees with Smith and colleagues [53], whom reported that when human DCs were co-incubated with the food related yeast *Kluyveromyces marxianus* a robust anti-inflammatory (*il10*) cytokine profile and subsequent Foxp3^+^Treg-cell type response was observed. In the current study compared to the control, we postulate that after 4 weeks of feeding the MsYF continuously the significant increases in gene expression of *il10* by 1.4-fold and *tgf*β by a 4-fold could be due to innate phagocytes orchestrating a cytokine milieu to induce the expansion of regulatory T-cells (Tregs). Interestingly, in mice that have a targeted mutation in the TGF-β gene develop severe multi-organ inflammation indicating a crucial role of TGF-β induced Treg induction [54–56].

The intestinal expression of interleukin 12 (*il12*) and interferon gamma (*ifnγ*) after 8 weeks of feeding the MsYF continuously regime was significantly elevated compared to the control by 2.7-fold and 1.5-fold, respectively. However, the lack of expression change for the transcriptional factor t-box (*t-bet*) suggests that there was no augmentation of Th1-like cell mediated immunity. Other studies using rainbow trout to model adaptive immune responses to bacterial infection with *Yersinia ruckerii* have demonstrated significant increases in the expression levels of both *t*-*bet* and *ifn*γ, suggestive of a Th1-like response [57,58]. In this context, the authors postulate that the up-regulation of both *il12* by 2.7-fold and *ifn*γ 1.5-fold after 8 weeks of feeding the MsYF continuously may be indicative of strengthening innate phagocytic and natural killer cell (NK) responses. In mammals, the co-operative effects of IL-12 and interleukin 18 (IL-18) in NK cell activation have been well characterised [59,60]. Moreover, in teleosts IL-18 homologues have been shown to be present and function in a similar way [61,62]. Therefore, the induction of NK cells could be driving the observed increase in gene expression of *ifnγ*, which in turn would activate inflammatory macrophage responses strengthening the innate response.

In teleosts, the differentiation and expansion of T-cell subsets is governed by the induction of lineage specific master transcription factors, including *t-bet* for Th1, gata binding protein 3 (*gata3*) for Th2, and RAR-related orphan receptor gamma (*rorγt*) for Th17 [63]. In the current investigation, the data suggests a potential Treg expansion through the significant expression in *gata3* after 8 weeks of feeding the MsYF (Fig 2B). In mice, *gata3* has been shown to control the fate and plasticity of Treg cells particular during inflammation [64,65]. Recently, Xu and colleagues concluded that high *gata3* expression converts functional Treg cells to Th1-Treg cells that can suppress a Th1-like cell response. Whereas, low *gata3* expression converts functional Treg cells to APC-like Treg cells that can modify the surveillance activities of antigen presenting cells such as macrophages, DCs and B-cells [66]. In the current study, there was no sign of inflammation at the morphometric level (Fig 1), despite this the high expression of 2.8 and 1.7 fold *gata3* suggests that supplementation of MsYF in the diet regardless of feeding strategy is potentially modulating T-cells responses to Th1-Treg to supress excessive inflammation. This warrants further investigation with possible targets for regulation and tolerance such as CD4^+^Foxp3^+^ regulatory T-cells would be important to help elucidate the mode of action of feeding MsYF to T-cell responses in teleosts.

In mice the delivery of antigens via goblet cell associated antigen passages (GAPs) is a major pathway for steady-state luminal antigen transfer to the LP-DCs in a manner capable of inducing antigen specific T cell responses [67,68]. In teleosts, associations of antigens with goblet cells in the second segment of the mid-intestine have demonstrated goblet cell-associated uptake [69,70]. In the current study, compared to control fish fed the MsYF supplemented diets demonstrated a significant increase in goblet cell density in both the anterior and posterior intestine. Particularly, in the posterior intestine there were highly significant elevations by 53.9% and 58% in MsYF groups. This data suggests the possibility that inclusion of MsYF could increase the capacity of goblet cell associated antigen uptake and surveillance of the underlying GALT tissue of rainbow trout. Data discerning histo-morphometry (Table 4), and scanning electron micrographs of fish fed the MsYF displayed no signs of cellular or enteritis like disruption where the epithelial surfaces appeared healthy with uniform enterocyte formations and densely packed microvilli (Fig 1).

In summary, there was no negative effect on growth performance when supplementing the basal diet with MsYF (Table 3). Feeding the MsYF to rainbow trout strengthened both the innate and adaptive response of the intestinal tissue with upregulation in both pro and anti-inflammatory effector cytokines alongside the elevated induction of transcripts for important transcription factors of adaptive responses. Furthermore, the increase in goblet cell density suggests that MsYF supplementation may increase the capacity for potential goblet cell associated antigen uptake. Rainbow trout suffer from a wide range of diseases caused by viral, bacterial and parasitic pathogens, including viral haemorrhagic septicaemia virus (VHSV) [70], enteric redmouth (ERM) disease [71] and proliferative kidney disease (PKD) [72], and so increased potential for antigen surveillance and uptake of GALT will be beneficial to the host. This preliminary evidence shows a positive step towards increasing the knowledge base of the mode of action of YCW extracts to enhance and strengthen the ability of innate immune cells of mucosal associated lymphoid tissues of rainbow trout.

## Supporting information

S1 File (XLSX)

S2 File (XLSX)
